# Engaging non-state providers towards PHC in South Asia: considerations for policymakers

**DOI:** 10.1016/j.lansea.2024.100454

**Published:** 2024-08-20

**Authors:** Zubin Cyrus Shroff, Anas Ismail, Kaosar Afsana, Manuj Weerasinghe, Krishna D. Rao

**Affiliations:** a Alliance for Health Policy and Systems Research, World Health Organization, Avenue Appia 20, Geneva, 1211, Switzerland; b BRAC James P Grant School of Public Health, BRAC University, Dhaka, Bangladesh; c Department of Community Medicine, Faculty of Medicine, University of Colombo, Sri Lanka; d Department of International Health, Johns Hopkins University, Baltimore, MD, USA

South Asia's large and heterogenous mix of non-state providers^e^ play a dominant role in the provision of curative healthcare services. Seventy percent of India's health workers (including 80% of its allopathic doctors) work in the private sector, with private providers having long been a major source of out-patient care.^1^ The private sector accounts for around two-thirds of available hospital beds in both Bangladesh and Nepal.^3^^,^^4^ While the public sector continues to be the dominant provider of hospital services in Sri Lanka, the recent growth of the private sector has led to over 50% of outpatient care being now provided outside government facilities.^5^ National health policies in the region have recognized the importance of engaging non-state providers as a necessary part of any long-term strategy to implement comprehensive Primary Health Care (PHC^f^) that underscores the achievement of Universal Health Coverage (UHC) in South Asia. However, bringing about the alignment of non-state providers with public health goals requires a paradigm shift away from the largely laissez-faire approach that has characterized the relationship between the government and private health sector in the region.^7^^,^^8^

Public Private Partnerships (PPP)^g^ are one approach that can play a role in facilitating the needed alignment, something that is increasingly recognized in the national health policies of countries in the region.^10^, ^11^, ^12^, ^13^ Bangladesh's Urban Primary Health Care Project that contracts NGOs to provide primary care in underprivileged communities; the Chiranjeevi Yojana in Gujarat, India that engages private providers to enhance access to institutional delivery among poorer groups; and Sri Lanka's engagement of private diagnostic and surveillance capacity to bolster malaria elimination under its Anti-Malaria Campaign are examples where South Asian countries have sought to leverage the comparative advantage of non-governmental actors to achieve specific health related goals.^14^, ^15^, ^16^ However, PPPs are not a panacea that will on their own overcome health system challenges. Further, when it comes to service delivery, studies on their effectiveness vis-à-vis the public sector show at best mixed results.^17^^,^^18^

Policymakers must therefore carefully consider whether a PPP is the most appropriate solution to address a given service delivery problem. This includes whether the non-state actor involved has any specific comparative advantage (such as an NGO being able to reach stigmatized or vulnerable groups). It also includes clearly examining the motivations of each actor (such as maximizing profit for the for-profit sector), clearly identifying potential conflicts of interest and assessing whether these can be managed to advance government policy goals.^19^^,^^20^ This is particularly the case for private for-profit providers and Finally, while ‘government failure’ in terms of the inability to efficiently manage financial and human resources is an oft used justification for PPPs, their effective design and implementation requires MoHs to develop new skills and capacities to carry out a range of functions that have not traditionally been within their remit.^18^^,^^21^ We argue that, while the public and private (and in particular the for-profit private) sectors have different goals, government leadership in the following four distinct areas is a minimum necessary for effective PPPs.

The first of these is for governments to clarify the goals of the PPP and the need to align funding, rules and policies with these goals. Reflecting on the experience of Bangladesh's Urban Primary Health Care Project, Islam et al. (2018) argue that the project's emphasis on cost recovery by NGOs undermined the project's stated objective of providing services to the most vulnerable. Similarly, the policy of awarding contracts to the lowest bidder without adequate weightage to service quality standards incentivized private actors to make unrealistically low bids to the detriment of the health of populations that they aimed to serve.^16^ Addressing this requires a clear diagnosis of the problem that the PPP seeks to address and empowering MoHs to engage with other relevant ministries including Ministries of Finance to enable policy coherence.^2^

Second is the strengthening of capacities at different levels of government to manage engagement with non-state providers. This includes capacities around writing and enforcing contracts.^2^ The absence of these capacities has been noted as a key impediment to the effective implementation of PPPs to achieve public health goals in several low-and-middle-income countries (LMICs).^16^^,^^17^ However, this is not an insurmountable challenge and the experience of Gujarat's Chiranjeevi Yojana makes clear how capacity strengthening efforts targeted at government officials enhanced their ability to effectively engage with the private sector in that setting.^22^

A third element is the need for governments to systematically generate and use evidence to inform PPP implementation. This requires investments in robust data and information systems, capacities to analyze data emanating from these systems as well as sensitizing policymakers and programme-implementers to the potential of evidence use.^23^^,^^24^ India's recent establishment of a Health Financing and Technology Assessment unit to inform its national health insurance programme is one example of the institutionalization of evidence use to inform the ongoing implementation of one of the world's largest PPPs for hospital services.^25^

Fourth, government policymakers have a key role in the development and maintenance of trusted relationships to advance the goals of the PPP.^18^ These relationships are essential enablers of open communication that is needed for effective implementation of the PPP as seen from the example of NGO engagement in Bangladesh's EPI programme.^26^ Further, as seen from Ghana's experience of engaging faith-based providers, engaging non-state providers as a strategic partner rather than a subordinate contractor, can facilitate the joint development of a long-term vision of the partnership and what role the different actors involved can play in realizing this vision.^2^

These four areas are deeply interconnected and there are synergies to be gained through coordinated action. Goal clarity, adequate financing and an enabling policy and regulatory environment are a *sine-qua-non.* In the absence of these, investments in government capacity will fail to have the desired effect on PPP success. Similarly, data that allow for continuous examination of PPP implementation and effectiveness and capacity to analyze such data are essential to inform ongoing implementation, course correction and adaptation of PPPs to ever evolving contextual changes. Finally, trust and relationships lie at the heart of meaningful partnerships, with the absence of trust serving as a major barrier to their effectiveness.^23^

Despite the preponderance of non-state providers in national health systems in South Asia, debates on their engagement by governments are particularly polarizing.^27^ We believe that viewing PPPs as a means to an end and not an end in and of themselves is an important first step. A second step is to realize that for-profit entities in particular, have goals that are different from those of public entities. There thus needs to be careful consideration of when a PPP is the most appropriate modality, as well as the implementation of measures to ensure that governments remain in the driving seat. Transparency around PPP objectives, making public the benefits accruing to different parties, ongoing evaluation of the added value of the PPP as well as clauses allowing for regular revision of PPP terms and conditions can facilitate the government's ability to play this role. It can also help improve low patient trust in the health system which is a challenge in several LMICs.^28^ Implementing these measures will require political will given the increasing influence of the private sector in influencing regulatory policies and practices in countries of the region over the past few decades.^29^ However, putting in place such measures is critical if governments are to harness the capacities and skills of non-state providers while ensuring that these contribute to public health goals and that health remains a public good.

## Contributors

ZCS and KDR drafted the manuscript with inputs from AI, KA and MW. All authors reviewed the final version.

## Ethical approval

Not needed as this is a comment and no human subjects were involved.

## Declaration of interests

We declare no competing interests. ZCS and AI are staff members of the World Health Organization. They themselves alone are responsible for the views expressed in the article, which do not necessarily represent the views, decisions, or policies of the World Health Organization.

## Footnotes

e We use the term non-state providers for not-for-profit and for-profit entities. This includes corporate hospitals, mission/faith based hospitals, non-government organizations, individual practices, or private pharmacies. Non-state providers (NSPs) can practice allopathic, traditional or faith-based systems of medicine. They can be formally trained or not; in several countries unqualified NSPs comprise a large portion of the health workforce.^1^^,^^2^

f We understand Primary Health Care (PHC) as ‘a whole-of-society approach with three components: (a) primary care and essential public health functions as a core of integrated health services; (b) multisectoral policy and action; and (c) empowered people and communities.^6^

g For the purpose of this commentary we define PPPs as “a long-term contract between a private party and a government agency, for providing a public asset or service, in which the private party bears significant risk and management responsibility”.^9^ We understand the private sector to include the not-for-profit sector in line with the definition in the footnote above.

## References

[1] Rao K.D., Shahrawat R., Bhatnagar A. (2016). Composition and distribution of the health workforce in India: estimates based on data from the National Sample Survey. WHO South East Asia J Public Health.

[2] Rao K.D., Paina L., Ingabire M.G., Shroff Z.C. (2018). Contracting non-state providers for universal health coverage: learnings from Africa, Asia, and Eastern Europe. Int J Equity Health.

[3] Rahman R. (2020). Shrinking the state: the rise of private sector healthcare in Bangladesh. J Int Dev.

[4] Adhikari R.P., Shrestha M.L., Satinsky E.N., Upadhaya N. (2021). Trends in and determinants of visiting private health facilities for maternal and child health care in Nepal: comparison of three Nepal demographic health surveys, 2006, 2011, and 2016. BMC Pregnancy Childbirth.

[5] Rannan-Eliya R.P., Ghaffoor A., Amarasinghe S. (2024). Sri Lanka's COVID-19 response and maintaining health services: implications for future pandemics. BMJ Glob Health.

[6] World Health Organization (2024). https://www.who.int/teams/integrated-health-services/clinical-services-and-systems/primary-care.

[7] Keshri V.R., Gupta S.S. (2019). Ayushman bharat and road to universal health coverage in India. J Mahatma Gandhi Inst Med Sci.

[8] Prinja S., Purohit N., Kaur N. (forthcoming) The state of primary healthcare in South Asia: Lancet Regional South East Asia.

[9] World Bank (2017).

[10] Ministry of Health and Population, Government of Nepal (2019). https://dohs.gov.np/national-health-policy2074/.

[11] Government of Peoples Republic of Bangladesh, Ministry of Health and Family Welfare (2011). http://www.mohfw.gov.bd/index.php?option=com_content&view=article&id=74&Itemid=9252.

[12] Government of India (2017). https://www.nhp.gov.in/nhpfiles/national_health_policy_2017.pdf.

[13] Ministry of Health, Government of Sri Lanka (2017). National Health Policy 2016 - 2025. https://www.health.gov.lk/wp-content/uploads/2022/08/2_National-Health-Policy-2016-2025_compressed.pdf.

[14] Yasobant S., Vora K.S., Shewade H.D. (2016). Utilization of the state led public private partnership program “Chiranjeevi Yojana” to promote facility births in Gujarat, India: a cross sectional community based study. BMC Health Serv Res.

[15] Fernando D., Wijeyaratne P., Wickremasinghe R. (2018). Use of a public-private partnership in malaria elimination efforts in Sri Lanka; a case study. BMC Health Serv Res.

[16] Islam R., Hossain S., Bashar F. (2018). Contracting-out urban primary health care in Bangladesh: a qualitative exploration of implementation processes and experience. Int J Equity Health.

[17] Odendaal W.A., Ward K., Uneke J. (2018). Contracting out to improve the use of clinical health services and health outcomes in low-and middle-income countries. Cochrane Database Syst Rev.

[18] Joudyian N., Doshmangir L., Mahdavi M., Tabrizi J.S., Gordeev V.S. (2021). Public-private partnerships in primary health care: a scoping review. BMC Health Serv Res.

[19] Brinkerhoff D.W., Brinkerhoff J.M. (2011). Public–private partnerships: perspectives on purposes, publicness, and good governance. Publ Adm Dev.

[20] Fanzo J., Shawar Y.R., Shyam T., Das S., Shiffman J. (2021). Challenges to establish effective public-private partnerships to address malnutrition in all its forms. Int J Health Pol Manag.

[21] Montagu D., Goodman C., Berman P., Penn A., Visconti A. (2016). Recent trends in working with the private sector to improve basic healthcare: a review of evidence and interventions. Health Pol Plann.

[22] World Health Organization (2007).

[23] Cross H.E., Sayedi O., Irani L., Archer L.C., Sears K., Sharma S. (2017). Government stewardship of the for-profit private health sector in Afghanistan. Health Pol Plann.

[24] Sheikh K., Agyepong I., Jhalani M. (2020). Learning health systems: an empowering agenda for low-income and middle-income countries. Lancet.

[25] Prinja S., Chugh Y., Gupta N., Aggarwal V. (2023). Establishing a health Technology assessment evidence ecosystem in India's pradhan mantri jan arogya Yojana. Health Syst Reform.

[26] Sajani T.T., Alo K., Aktaruzzaman S.A. (2014). Public private partnership (PPP) in health sector of Bangladesh. Anwer Khan Mod Med Coll J.

[27] Sengupta A., Nundy S. (2005). The private health sector in India. BMJ.

[28] Kruk M.E., Gage A.D., Arsenault C. (2018). High-quality health systems in the Sustainable Development Goals era: time for a revolution. Lancet Global Health.

[29] Hunter B.M., Murray S.F., Marathe S., Chakravarthi I. (2022). Decentred regulation: the case of private healthcare in India. World Dev.

